# Distribution of *Burkholderia pseudomallei* within a 300-cm deep soil profile: implications for environmental sampling

**DOI:** 10.1038/s41598-022-12795-0

**Published:** 2022-05-23

**Authors:** Khemngeun Pongmala, Alain Pierret, Priscia Oliva, Anne Pando, Viengmon Davong, Sayaphet Rattanavong, Norbert Silvera, Manophab Luangraj, Laurie Boithias, Khampaseuth Xayyathip, Ludovic Menjot, Melina Macouin, Emma Rochelle-Newall, Henri Robain, Amphone Vongvixay, Andrew J. H. Simpson, David A. B. Dance, Olivier Ribolzi

**Affiliations:** 1 GET, Université de Toulouse, CNRS, IRD, UPS, Toulouse, France; 2 Faculty of Environmental Sciences, National University of Laos, Vientiane, Lao PDR; 3 Institut de Recherche Pour le Développement (IRD), IEES-Paris UMR 242, Sorbonne Université C/o Department of Agricultural Land Management (DALaM), Vientiane, Lao PDR; 4 Lao-Oxford-Mahosot Hospital-Wellcome Trust Research Unit (LOMWRU), Microbiology Laboratory, Mahosot Hospital, Vientiane, Lao PDR; 5 Institute of Ecology and Environmental Sciences of Paris (iEES-Paris), Sorbonne Université, Univ Paris Est Creteil, IRD, CNRS, INRA, 4 place Jussieu, 75005 Paris, France; 6 Faculty of Engineering, National University of Laos, Vientiane, Lao PDR; 7 Centre for Tropical Medicine and Global Health, Nuffield Department of Medicine, University of Oxford, Oxford, UK; 8 Faculty of Infectious and Tropical Diseases, London School of Hygiene and Tropical Medicine, London, UK

**Keywords:** Infectious diseases, Soil microbiology

## Abstract

The environmental distribution of *Burkholderia pseudomallei*, the causative agent of melioidosis, remains poorly understood. *B. pseudomallei* is known to have the ability to occupy a variety of environmental niches, particularly in soil. This paper provides novel information about a putative association of soil biogeochemical heterogeneity and the vertical distribution of *B. pseudomallei*. We investigated (1) the distribution of *B. pseudomallei* along a 300-cm deep soil profile together with the variation of a range of soil physico-chemical properties; (2) whether correlations between the distribution of B. pseudomallei and soil physico-chemical properties exist and (3) when they exist, what such correlations indicate with regards to the environmental conditions conducive to the occurrence of *B. pseudomallei* in soils. Unexpectedly, the highest concentrations of *B. pseudomallei* were observed between 100 and 200 cm below the soil surface. Our results indicate that unravelling the environmental conditions favorable to *B. pseudomallei* entails considering many aspects of the actual complexity of soil. Important recommendations regarding environmental sampling for *B. pseudomallei* can be drawn from this work, in particular that collecting samples down to the water table is of foremost importance, as groundwater persistence appears to be a controlling factor of the occurrence of *B. pseudomallei* in soil.

## Introduction

*Burkholderia pseudomallei* is a Gram-negative bacterium endemic in the tropics, particularly northern Australia and South-East Asia, that causes melioidosis and was first recognized in 1911 in Burma by Alfred Whitmore and his assistant C. S. Krishnaswami^1^. Melioidosis affects both humans and animals and suspected infection routes include contact of lesions with soil and surface waters, as well as ingestion and inhalation of contaminated water and aerosols^2^. The global burden of melioidosis is estimated to be of the order of 165,000 cases per year, more than half being fatal^3^. The global burden of melioidosis worldwide is estimated to be 4.6 million disability-adjusted life years (DALYs), which represents a major public health problem, and the incidence and mortality rates are twice as high in men than women^4^. Human melioidosis cases tend to be related to the presence of *B. pseudomallei* in soil^5^.

The environmental distribution of *B. pseudomallei* and the true burden of melioidosis remain poorly understood^5,6^. The presence of *B. pseudomallei* is reportedly correlated with environmental factors such as climate, as well as soil physico-chemical and biological factors^7–9^. Extreme weather such as heavy rainfall events and flooding are commonly associated with both increased environmental detection of *B. pseudomallei*^10–12^ and increased incidence of melioidosis, although it is unclear what precise mechanism drives this behaviour of the organism.

Some studies have highlighted the fact that landscape features may influence the occurrence and spread of *B. pseudomallei*^13–15^ and that anthropic factors, particularly land use, can induce shifts in the microbial community structure that may affect its presence in soils^13,14^. In a rice paddy field in central Lao People’s Democratic Republic (Laos), *B. pseudomallei* was found to occur preferentially in soil with high moisture content but low organic matter and nitrogen contents^16^. Likewise, based on a survey of 61 rice fields in Thailand, Hantrakun et al.^17^ found that *B. pseudomallei* was associated with nutrient-depleted soils and suggested that agricultural practices that induce soil degradation may increase the presence and amount of *B. pseudomallei* in endemic areas.

Not unexpectedly, moisture is a parameter of prime importance to the survival of *B. pseudomallei*^18^. *B. pseudomallei* can survive over a year in soil at a 20% moisture content, but only 30 days in dry soil^19^. In the field, *B. pseudomallei* has nevertheless been identified in humid habitats of otherwise arid environments^20^ and in places where dry conditions prevail after heavy rains^21,22^.

Some micro-nutrients, such as iron, which is suspected to play an important role in the expression of respiration enzymes^23^, are likely favorable to the presence of *B. pseudomallei* ^24^. *B. pseudomallei* is nevertheless known to adapt to various environmental stresses such as salinity, oxidative stress or low iron content and to occupy a variety of ecological niches, particularly in soil^7,17^. There is potential for several environmental reservoirs to offer niches for *B. pseudomallei*^25^, from which the bacteria can be disseminated over large distances including through hydrological processes^13^ such as soil surface runoff^26^, thus increasing the risk of human infection.

Attempts to characterize such niches, particularly in soil, remain few and lacking in detail (or undertaken at a relatively coarse scale), despite well documented evidence that soil encompasses intricately nested biotic and abiotic components and functions, thus forming a complex ecosystem^27^. Up to now, environmental surveys of *B. pseudomallei* have relied on the recommendations of the Detection of Environmental *B. pseudomallei* Working Party (DEBWorP)^28^ which suggest collecting a minimum of 100 individual soil samples, taken from point locations 2.5–5 m apart from an area of about 50 × 50 m, at a depth of 30 cm below the soil surface. While these recommendations were established based on an exhaustive analysis of environmental studies on *B. pseudomallei*, they did not include any consideration of the intrinsically universal arrangement of soils as so-called profiles^29^ nor the associated processes of soil formation (pedogenesis). Soil profiles consist, from ground surface litter to bedrock, of superimposed and laterally homogeneous layers, known as soil horizons. Along the vertical axis, soil horizons of a given profile differ in their biogeochemical makeup at scales of a few centimeters to tens of centimeters, while within each horizon, biogeochemical conditions can also be very different in ways of utmost relevance to microbial activity (soil structure and microstructure^30^). Current knowledge of soil microbiology relies on sampling of the topsoil layers, based on the assumption that these layers concentrate the majority of microbial biomass, activity and diversity. However, recent studies indicate that some microbial taxa preferentially adapted to low-nutrient availability are more abundant in deep soils, suggesting that the depth-wise variability of soil physico-chemical properties may be the most important factor shaping the structure of soil microbial communities^31,32^.

While information about the vertical distribution of *B. pseudomallei* in soils is rare, a few studies have suggested that *B. pseudomallei* may occur at soil depths > 30 cm. For example, a survey of 360 sampling sites in Hainan, China, consistently found *B. pseudomallei* in soil samples taken at depths of 30–60 cm and suggested that documenting soil heterogeneity would help better to understand the distribution and prevalence of this pathogen in soil^33^. Studies conducted in Myanmar, in Northeastern Thailand and in the plain of Vientiane, Lao PDR, also indicated that *B. pseudomallei* can be more abundant at soil depths of 90 cm than nearer the soil surface^16,34,35^.

In an attempt to provide new detail about the heterogeneity of the distribution of *B. pseudomallei* within soils, particularly along the vertical axis, and to document whether this putative heterogeneity covaries with soil biogeochemical properties, the work presented in this paper aimed to investigate (1) the distribution of *B. pseudomallei* along a 300-cm deep soil profile together with the variation of a range of soil physico-chemical properties; (2) whether correlations between the distribution of *B. pseudomallei* and soil physico-chemical properties exist and (3) when they exist, what such correlations indicate with regards to the environmental conditions conducive to the presence of *B. pseudomallei* in soils.

## Results

### Distribution of ***B. pseudomallei*** along a soil profile in a lowland paddy rice field (Fig. 1)

**Figure 1:**
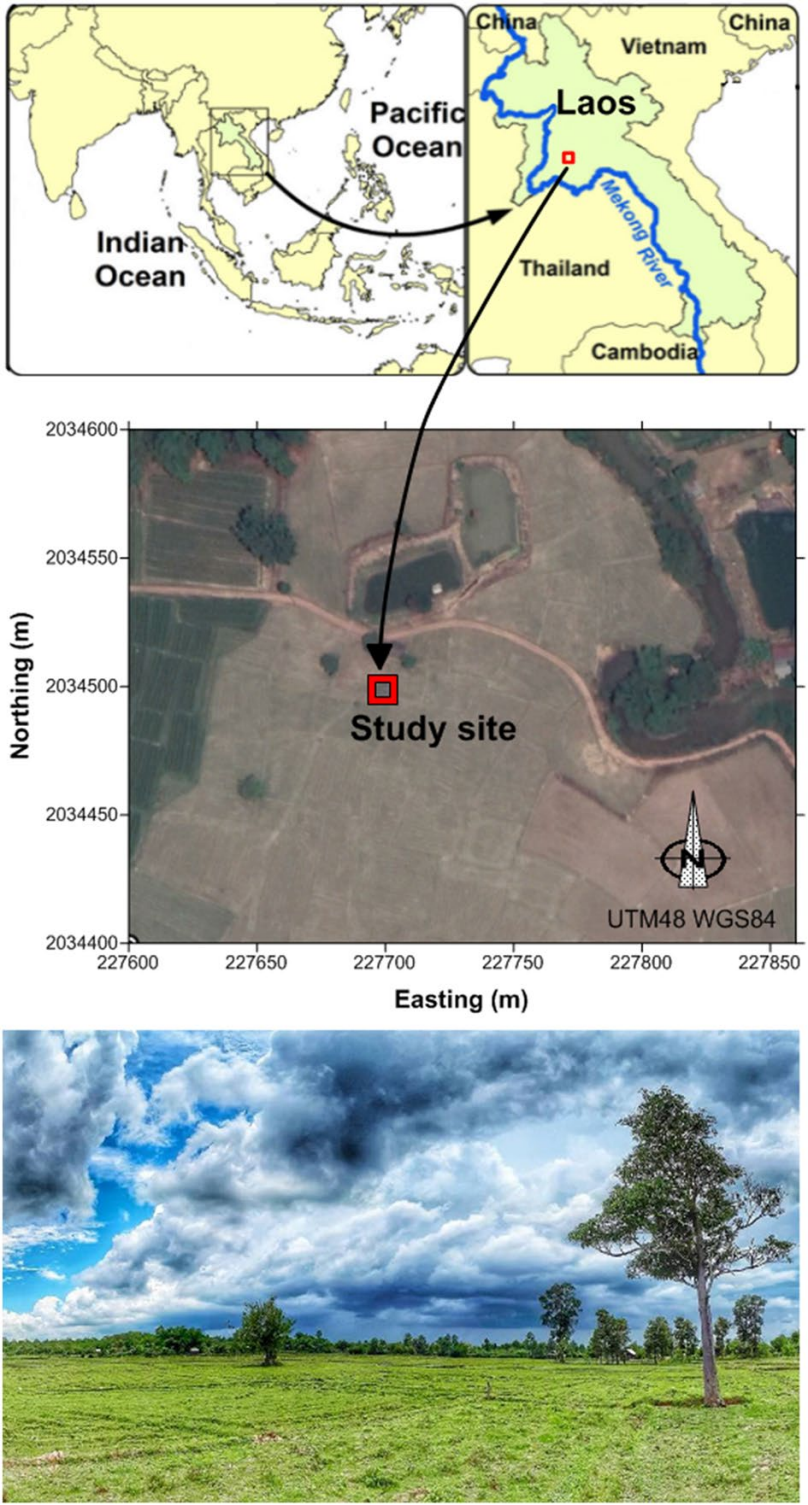
Study site: (**a**) location of the site in northern Laos; (**b**) situation within the cultivated plain of the Nabone village (Satellite image 2019-02-22, Image © 2021 Maxar Technologies, Google Earth); (**c**) photograph of the paddy rice field where the soil profile was observed, monitored, and sampled. This figure was created using Surfer^®^ 11 (Golden Software Inc.).

**Figure 2:**
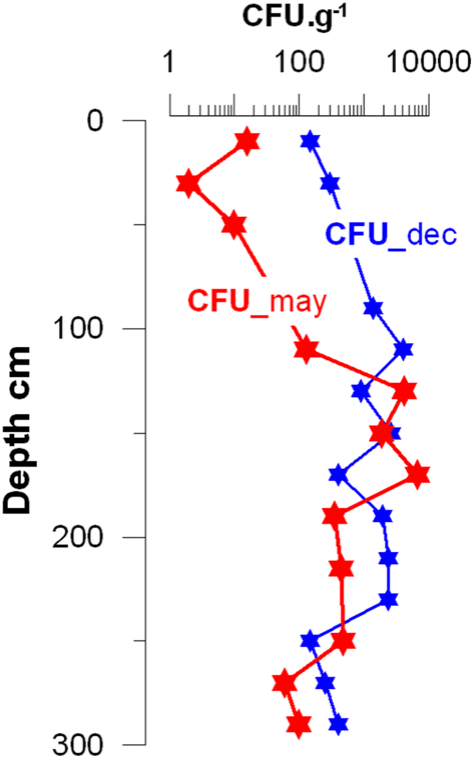
Semi-quantitative colony forming unit (CFU) counts of *B. pseudomallei* (CFU g^−1^) along the 300-cm deep profile, in May (CFU_may) and December (CFU_dec) 2018. *B. pseudomallei* was not detected at 80-cm and 230-cm in May, and at 50-cm in December.

Semi-quantitative cultures of soil samples taken along a profile in May 2018 allowed the detection of *B. pseudomallei* at nearly all depths except for 50, 80 and 230 cm. Although counts averaged 1179 CFU g^−1^ over the whole profile, values varied drastically spanning four orders of magnitude, from 1 to 12,600 CFU g^−1^, with the highest *B. pseudomallei* concentrations between the soil depths of 110 cm and 250 cm. The maximum *B. pseudomallei* concentration was observed at 170 cm soil depth (Fig. 2).

An additional 300-cm vertical profile collected in December 2018 revealed that, below 100 cm, depth-wise *B. pseudomallei* concentrations were of the same order of magnitude as that observed in May 2018. In the first meter of the profile, CFU counts were more dissimilar between the two sampling dates, which might be related to the re-wetting of the profile in December, following the end of the rainy season, in line with the previous results of Manivanh et al.^16^ (Fig. 2).

### Morpho-pedological features

**Figure 3:**
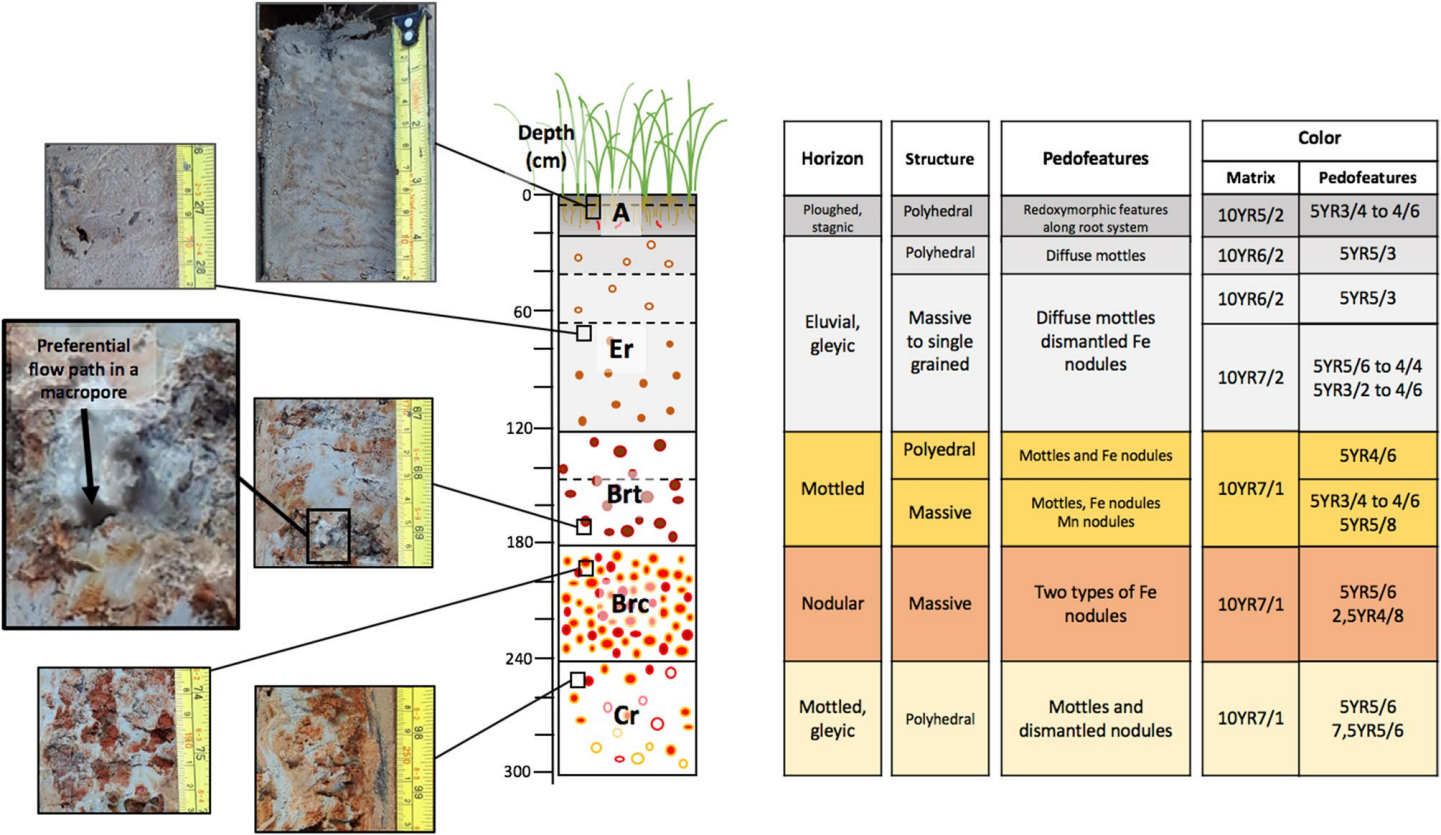
Morpho-pedological features of the studied soil profile: center, schematic of the soil log showing the vertical succession and thickness of the main horizons (i.e. A, Er, Brt, Brc and Cr); left, pictures showing colored features mainly related to iron redistribution, and a preferential water flow path in a macropore; right, synthesis table of the soil horizons structure, pedofeatures and colors (Munsell color chart^37^).

The pedological description (Fig. 3) revealed that the studied soil belongs to the gleyic plinthic Acrisol type according to the international soil classification^29^. Such a soil is characterized by intense clay leaching processes inducing clay depletion in surface horizons and clay accumulation in deeper horizons (i.e., argic horizon). Morphological features indicate that the soil is highly weathered and has experienced intense oxidation–reduction processes, as evidenced by the dismantling of iron-containing nodules. In the argic horizons, soil shows non-homogeneous porosity features with visible preferential pathways (Fig. 3, picture taken at 169 cm depth). From the surface to the depth of 300 cm, the soil profile can be divided into 5 main soil horizons, with some sub-horizons being distinguished based on variations in pedofeatures (Fig. 3).

- The first soil horizon mainly resulted from anthropo-pedogenetic^36^ processes (hydragric A-horizon, 0–20 cm), had a sandy-loam texture and a dark greyish brown colour (10YR5/2) related to high organic matter content and displayed ploughing and stagnic traits. Redoximorphic features displaying specific reddish colours from 5YR3/4 to 4/6 were observed along biopores and root channels;
- The underlying albic horizon (Er-horizon), extending between depths of 20–120 cm was characterized by a pronounced sandy texture with sand contents of > 70% between 30 and 110 cm, and a lighter grey colour (10YR6/2 to 10YR7/2) corresponding to a lower organic matter content than in the A horizon. Iron nodules occurred increasingly towards the lower part of the horizon with colours from 5YR5/3 to 5YR3/2;
- The third soil horizon (Brt horizon) is an argic B horizon which extends between the depths of 120 and 180 cm had a high clay content, reaching a maximum of 46% at 170 cm, a light grey matrix colour of 10YR7/1 and included increasing amounts of iron and manganese nodules with colours mainly yellowish red (5YR4/6) giving it an apparent light yellow–red colour. Nodules in this horizon showed signs of dismantling;
- The fourth horizon (Bcr horizon) is a plinthic nodular horizon, extending from 180 to 240 cm and characterized by the presence of two types of iron nodules with notable colour differences (2.5YR4/8 and 5YR5/6), in amounts even higher than in the Brt horizon, resulting in an even more pronounced orange overall coloration despite of similar matrix colour (10YR7/1). Clay content decreased regularly from the top to the bottom of this horizon, mirrored by increasing amounts of sand;
- Finally, between 240 and 300 cm lay the last horizon (Cr-horizon) of sandy-clay-loam texture with overall a much lighter grey-white colour due to lower content of apparently fragmented and dismantled iron nodules.

### Physico-chemical parameters

**Figure 4:**
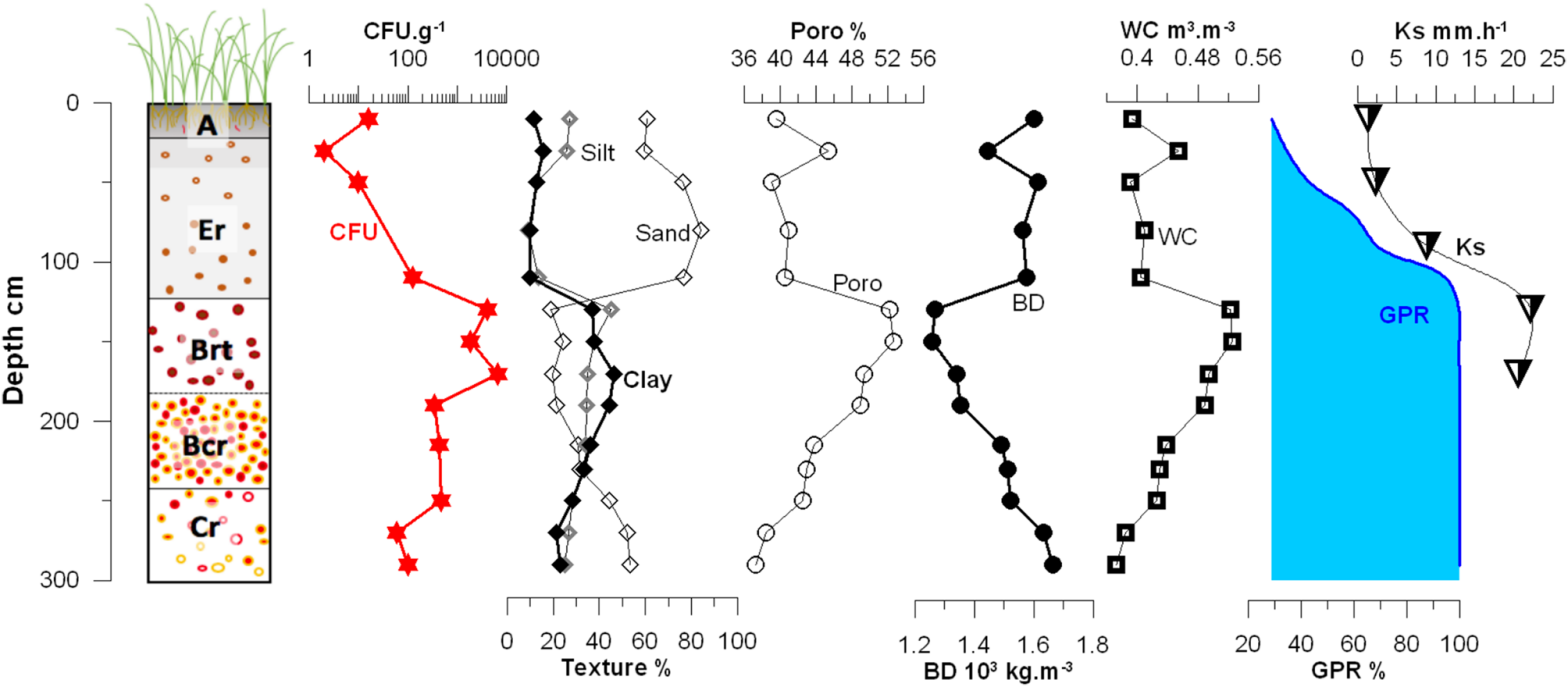
Soil log showing the main pedological horizons (i.e. A, Er, Brt, Bcr and Cr—A = surface (topsoil) horizon; E = eluvium soil horizon (horizon leached of its mineral and/or organic content, B = subsoil horizon (layers which are significantly altered by pedogenesis, mostly with the formation of iron oxides and clay mineral); C = parent material horizon (layer marginally affected by pedogenesis). When indicated, abbreviations for master soil horizon subdivisions are: r = weathered or soft; t = accumulation of silicate clays; c = concretions or hard nodules), semi-quantitative colony forming unit (CFU) counts of *B. pseudomallei* (CFU g^−1^), and soil physical parameters along the 300-cm deep profile: texture (i.e. percentages of silt, sand and clay), porosity (Poro), bulk density (BD), volumetric water content (WC), groundwater persistence rate (GPR) and saturated hydraulic conductivity (Ks).

Particle size analysis revealed that sand fraction (60–84%) prevailed in the surface and subsurface horizons (A and Er) to a depth of 110 cm (Fig. 4). Variations in particle size distribution with depth indicated a depletion of both clay and silt in the surface horizons (10–16% for clays and 10–26% for silts) which resulted in an enrichment of the deeper B horizons (33–44% clays) from 120 to 230 cm. Such a particle size evolution with depth, a typical pattern for soil experiencing clay leaching such as Acrisol, confirms the horizon succession that our pedological observations revealed, in particular, the presence of argic horizons between 120 and 240 cm depth. Below 230 cm, the decrease in clay content (28–21%) indicated the transition between the argic horizon and the saprolite (Cr horizon).

Variations in porosity with depth indicated that the highest porosity value (~ 52%) is in the argic horizon (Fig. 4). If textural micropores probably constitute the major fraction of total porosity in this horizon, structural macropores, revealed by field observations, are also part of the soil porosity. This well-developed network of macropores allows preferential flow pathways, hence likely explains the higher hydraulic conductivity values measured in the third soil horizon compared to the first and the second ones (Fig. 4).

**Figure 5:**
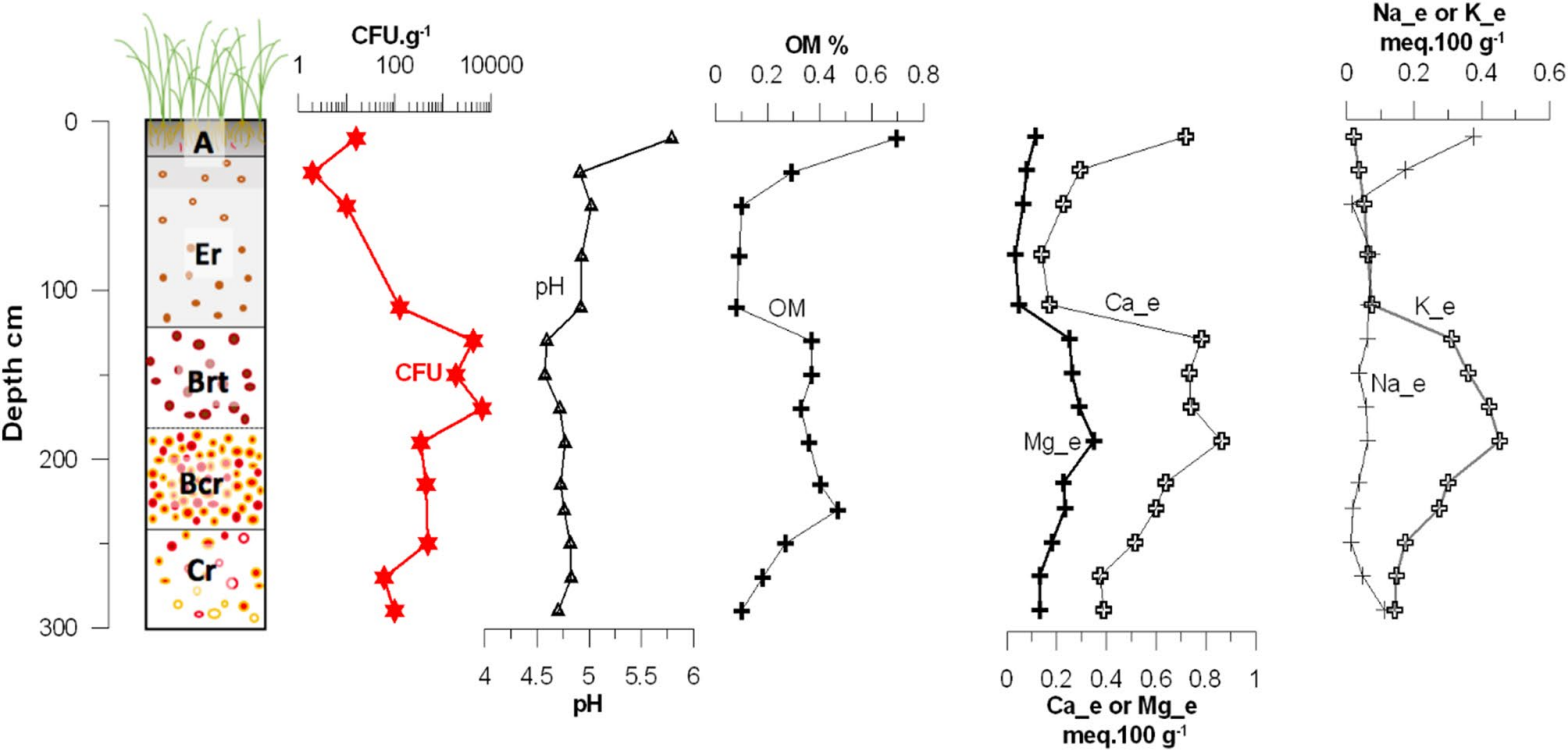
Soil log showing the main pedological horizons (i.e. A, Er, Brt, Bcr and Cr—see caption of Fig. 4 for meaning of abbreviations), semi-quantitative colony forming unit (CFU) counts of *B. pseudomallei* (CFU g^−1^), and soil physico-chemical parameters along the 300-cm deep profile: pH, organic matter content (OM), exchangeable magnesium (Mg_e), exchangeable calcium (Ca_e), exchangeable sodium (Na_e) and exchangeable potassium (K_e).

Organic matter (OM) content was low (0.1–0.7%) in the studied soil profile, with the highest values observed in the surface horizon (Fig. 5). However, the OM profile suggested a relative enrichment of organic compounds in the argic horizons (about 0.37–0.47% of OM content between 120 and 240 cm). The origin of such organic matter enrichment at depth cannot be fully determined based on the analysis made in this study^38^. It can either result from the migration and incorporation of mobile organic compounds from the Er horizon toward the B horizons, similarly to the clay and silt fractions, or from the persistence of organic matter compounds in relation with past ecosystem functioning prior to rice cultivation (e.g., forest ecosystem).

Soil pH was acidic with pH values ranging from 4.58 to 5.02 (Fig. 5) with the exception of the surface horizon being less acidic (pH = 5.79) probably due to higher OM in the A soil horizon (Fig. 5) and the temporary puddling of the rice crop^39^. The very CEC of this soil (i.e., < 2.5 meq 100 g^−1^) coupled with a relatively high exchange acidity (between 20 and 35% of the total CEC) except for the surface horizon, creates favorable conditions for clay leaching. Ca_e, Mg_e, Na_e and K_e are low and reach a maximum value of 1.7 meq 100 g^−1^ at 290 cm depth (Fig. 5). These results are in line with the previous study of Matsuo et al.^40^ for soils from the Phonghong district, Laos.

**Figure 6:**
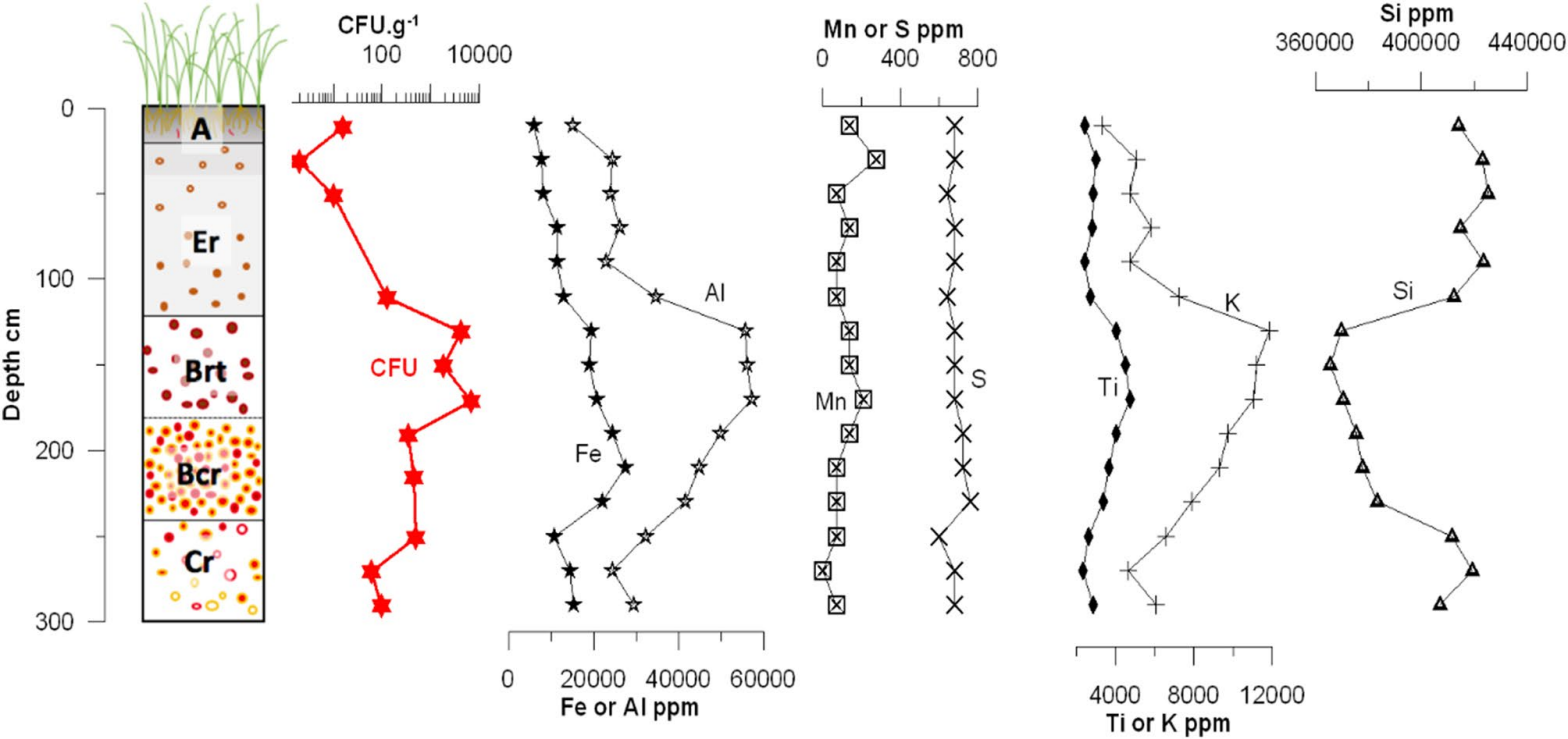
Soil log showing the main pedological horizons (i.e. A, Er, Brt, Bcr and Cr—see caption of Fig. 4 for meaning of abbreviations), semi-quantitative colony forming unit (CFU) counts of *B. pseudomallei* (CFU g^−1^), and elemental concentration along the 300-cm deep profile: iron (Fe), aluminum (Al), manganese (Mn), sulfur (S), titanium (Ti), potassium (K), silicium (Si).

Major element concentrations showed inverted profiles for the major elements Al and Si (Fig. 6); Si was depleted in the argic horizons in contrast with Al and K that displayed significant increases. These results are consistent with clay leaching processes occurring in this soil and the clay rich nature of horizons between 120 and 240 cm. Fe concentration gradually increases from surface down the soil profile, reaching a maximum at a depth of 215 cm, corresponding to a deep nodular horizon.

Parallel to major element concentrations, mineralogical investigations (powder X-ray diffraction patterns) showed that quartz was the dominant mineral at the whole soil profile scale, regardless of the horizons (Fig. SI1). However, not unexpectedly, the intensity of peaks corresponding to clay minerals increased in argic horizons samples. X-ray diffraction analysis of the mineralogical composition of the < 2 µm revealed a similar clay minerals assemblage for all soil horizons, consisting of Mg vermiculite, chlorite, illite, kaolinite, and interstratified phases of illite and vermiculite type (Fig. SI2). Mg vermiculite and chlorite phases are common in paddy field soils prone to redox processes. Indeed, mineralogical transformations can occur in such soils such as chloritization and the loss of K from micaceous clays^41^. Iron rich phases were only apparent in powder X-ray diffraction patterns from 120 cm depth and were mainly ferrihydrite and goethite for the fine earth fraction. Nodules were composed of hematite. Specific analysis of the < 2 µm clay fraction also revealed the existence of lepidocrocite. Both ferrihydrite and lepidocrocite are iron bearing phases that form during in situ soil processes and which are commonly observed within redoximorphic features of rice paddy fields. The observed mineralogical assemblages for the studied soil are similar to those observed by Egashira et al.^42^ from soils developed on similar geological substrate in the Vientiane plain.

### Groundwater persistence

The average groundwater persistence rate^43^ (GPR), defined as the number of days during which soil was saturated with water, divided by the number of days in a year, was calculated (Fig. 4) based on the data of groundwater from both automatic and manual measurement within 1 year (May 2018–May 2019). GPR was equal to 100% at all soil depths > 120 cm. Groundwater depths varied between a minimum of less than 15 cm between June and September and a maximum of 175 cm at the end of the dry season (May). GPR was at least 99% in the 130–290 cm depth range where the highest concentrations of *B. pseudomallei* were measured.

### Statistical analysis

**Figure 7:**
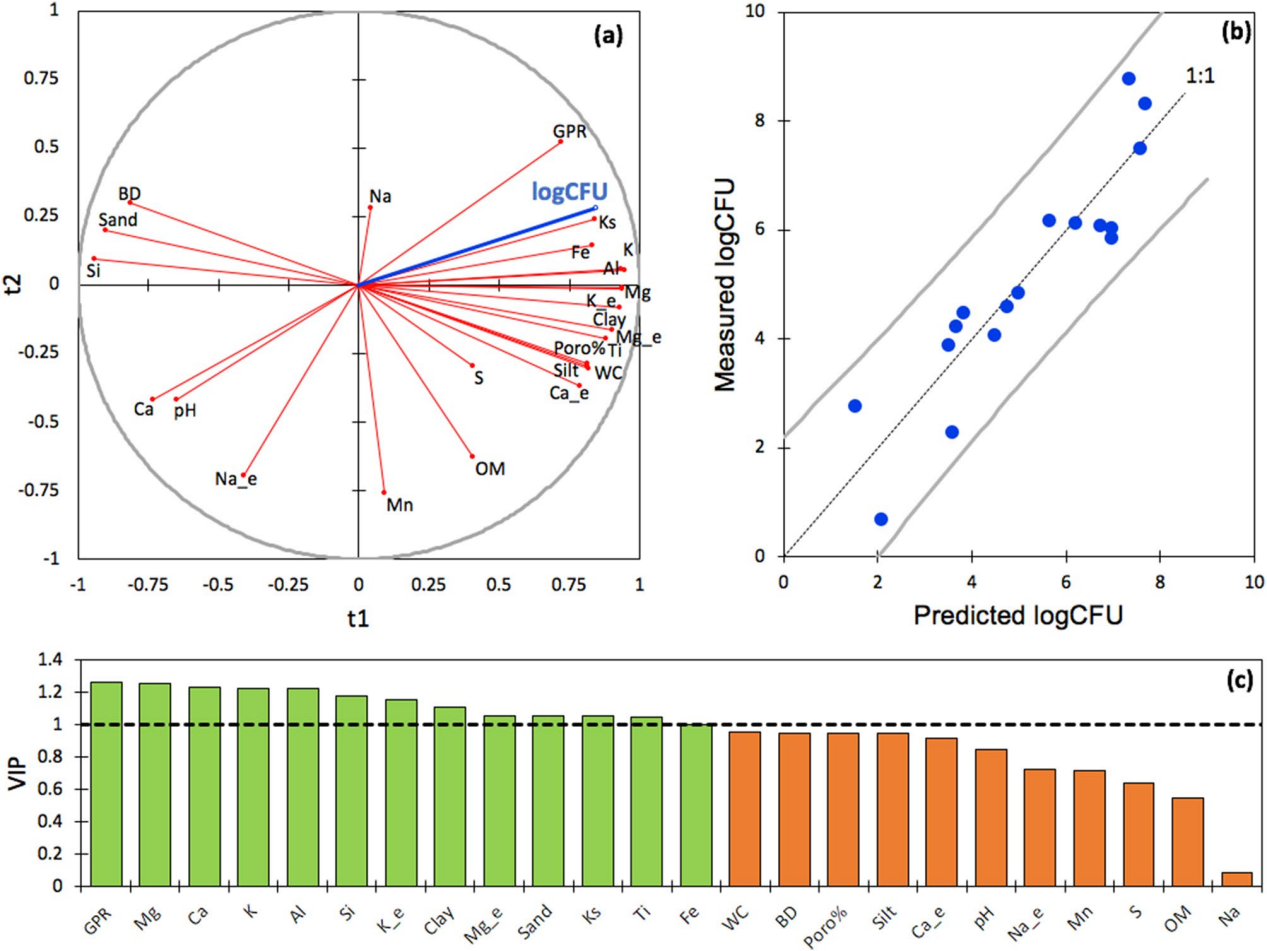
Partial Least Squares Regression (PLS-R) analysis where the natural logarithm of the semi-quantitative colony forming unit (CFU) counts of *B. pseudomallei* (logCFU) are compared with soil physico-chemical variables: (**a**) logCFU in the correlation circle with the 24 soil parameters; (**b**) Measured and predicted logCFU using the PLS-R model; (**c**) Variable importance for the projection (VIP) score plot of the 24 soil physico-chemical variables. *Fe* total iron concentration, *Al* total aluminum concentration, *Mn* total manganese concentration, *S* total sulfur concentration, *Ti* total titanium concentration, *K* total potassium concentration, *Si* total silicium concentration; pH, *OM* organic matter content, *Mg_e* exchangeable magnesium fraction, *Ca_e* exchangeable calcium fraction, *Na_e* exchangeable sodium fraction, *K_e* exchangeable potassium; silt, sand and clay textural fractions; *Poro* soil porosity, *BD* bulk density, *WC* volumetric water content, *GPR* groundwater persistence rate, *Ks* saturated hydraulic conductivity.

The PLS-R analysis considering all soil depths from 10 to 290 cm and all measured variables (Fig. 7), including saturated hydraulic conductivity (Ks), with values interpolated to 290 cm slightly outperformed a first PLS-R that did not take Ks into account. This confirms the importance of Ks as an explanatory variable throughout the profile and had a cumulated Q^2^ of 0.70 and 0.64 on the first two components, respectively, indicating an overall good quality of the fit. The cumulated R^2^Y and R^2^X also ranged from 0.60 to 0.79, indicating that the first two components summarize well both the Xs and the Ys. The best explanatory variables of this first PLS regression were: GPR, Ks, Ca, Al, Si, Clay, Mg, K, Mg_e and K_e, Sand, Ti and Fe.

Overall, there was a contrast between the Spearman’s rank-order correlations computed considering on the one hand, the full profile (10–290 cm) and on the other hand, the 10–170 cm range only (i.e. the depth to which Ks was measured) (Table 1). More correlations (18 vs 13) and at a higher significance level (17 vs 6 correlations at p < 0.01) were found when considering the full soil profile (10–290 cm) compared to the 10–170 cm range only. CFU counts from semi-quantitative cultures of *B. pseudomallei* thus appeared significantly correlated with:

- clay (ρ = 0.71; p = 0.002); silt (ρ = 0.76; p < 0.001); and sand contents (ρ = − 0.76; p < 0.001);
- Fe (ρ = 0.68; p = 0.003); Mg (ρ = 0.90; p < 0.0001); Ti (ρ = 0.62; p = 0.009); Al (ρ = 0.87; p < 0.0001); K (ρ = 0.87; p < 0.0001); Si (ρ = − 0.86; p =  < 0.0001) and Ca (ρ = − 0.78; p = 0.0003);
- exchangeable Ca (ρ = 0.63; p = 0.008); Mg (ρ = 0.72; p = 0.0013); and K (ρ = 0.86; p < 0.0001);
- soil bulk density (BD) (ρ = − 0.66; p < 0.005), Poro (ρ = 0.66; p < 0.005) and GPR (ρ = 0.60; p < 0.05);
- soil pH (ρ = − 0.80; p < 0.001).

**Table 1:** Correlation (Spearman r coefficient) between the semi-quantitative colony forming unit (CFU) counts of *B. pseudomallei* (CFU g^−1^) and soil physico-chemical characteristics at the full soil profile depth (10–290 cm) and the maximum depth that the saturated hydraulic conductivity was measured (10–170 cm).

Physico-chemical parameters	Soil profile depth	
	10–290 cm	10–170 cm
pH	− 0.80***	− 0.69*
OM	0.37	0.10
Ca_e	**0**.63**	0.43
Mg_e	**0**.73**	0.43
K_e	0.86***	0.95***
Na_e	− 0.42	− 0.30
Sand	− 0.76***	− 0.41
Silt	0.76***	0.49
Clay	0.71**	0.36
BD	− 0.66**	− 0.65*
Poro	0.66**	0.65*
WC	0.66**	0.65*
GPR	0.61*	0.95***
Na	− 0.03	− 0.12
Mg	**0**.90***	0.94***
Al	**0**.87***	0.78*
Si	− 0.86***	− 0.76*
S	0.24	0.19
K	0.87***	0.78*
Ca	− 0.78***	− 0.87**
Ti	0.62**	0.45
Mn	0.01	0.04
Fe	0.68**	0.94***
Ks		0.94***

Significance level: ∗∗∗p < 0.001, ∗∗p < 0.01, ∗p < 0.05.

Listed are pH; organic matter content (OM, %); exchangeable calcium (Ca_e, meq 100 g^−1^); exchangeable magnesium (Mg_e, meq 100 g^−1^); exchangeable potassium (K_e, meq 100 g^−1^); exchangeable sodium (Na_e, meq 100 g^−1^); silt, sand and clay textural fractions (%); bulk density (BD, g cm^3^); soil porosity (poro, %); volumetric water content (WC, m^3^ m^−3^); groundwater persistence rate (GPR, %); iron content (Fe, ppm); aluminum content (Al, ppm); manganese content (Mn, ppm); sulfur content (S, ppm); titanium content (Ti, ppm); potassium content (K, ppm); silicium content (Si, ppm); saturated hydraulic conductivity (Ks, mm h^−1^).

Additionally, when considering the soil profile from 10 to 170 cm only, i.e., the maximum depth at which Ks was measured, semi-quantitative cultures of *B. pseudomallei* were significantly correlated with Ks (ρ = 0.94; p < 0.0001).

## Discussion

The soil physico-chemical parameters presented in this paper that appear to be of importance with regards to the the vertical distribution of *B. pseudomallei* are those with the highest VIP values in the PLS-R, namely, GPR, Mg, Ca, K, Al, Si, K-e, Clay, Mg_e, Sand, Ks, Ti and Fe. The group of chemical elements thus outlined is consistent with the apparent importance of clay minerals. Otherwise, GPR and soil structure (Ks—hence aerobic conditions) appear to be the parameters that are most significantly correlated with the observed vertical distribution of *B. pseudomallei*.

Apart of considerations related to the ecology of *B. pseudomallei*, an essential contribution of this work is the reminder that soils are not restricted to a few centimeters below the surface and that their physico-chemical and mineralogical properties vary with depth, consistent with changes in morphopedological features. Recent studies of *B. pseudomallei* emphasize that different interacting factors (climate, physico-chemical and biological condition) in soil and groundwater may influence the occurrence and spread of the organism. However, most investigations of the ecology of *B. pseudomallei* have so far overlooked altogether the fact that soils do not correspond to large volumes of homogeneous material below the ground surface but are instead one of the most complex ecosystems on Earth. As a direct consequence of the over-simplistic representation of soils that emerges from the literature on the ecology of *B. pseudomallei*, the overwhelming majority of the thousands of samples that have been specifically collected to detect this bacterium were collected from a depth of at most 30 cm, and very occasionally up to 90 cm. As a first attempt to take a different look at the distribution of *B. pseudomallei* in soil, here we have, for the first time, examined the semi-quantitative distribution of *B. pseudomallei* along a 300-cm deep soil profile, in relation to environmental covariates related to soil physico-chemical properties.

Indeed, soils generally consist of successive layers (i.e.,horizons), each having their own biogeochemical makeup and dynamics. Within each horizon, the arrangement of solid particles creates several levels of nested structures, representing a myriad of potential niches for communities of living organisms, including (1) bacteria, fungi, protozoa and nematodes at scales < 100 μm (2) acari, springtails, diplura, symphylans and enchytraeids at scales > 100 μm and < 2 mm and (3) mollusc, spiders, insects, earthworms at scales > 2 mm^44^.

### Soil texture

Along the studied soil profile, soil texture, which reflects the relative proportions of sand, silt and clay, varied substantially as a function of soil depth, as a consequence of clay leaching processes. Soil texture was predominantly sandy in the first meter of the profile, then markedly clayey between 120 and 210 cm and finally gradually sandier again deeper in the profile. Such variations in soil texture were well correlated with *B. pseudomallei* counts, with fine-textured material (and clay (< 2 μm) and silt (2–50 μm)) and coarser material (sand (0.05–2.0 mm)) positively and negatively correlated with *B. pseudomallei* counts, respectively. Information suggesting the existence of a correlation between soil texture and the presence of *B. pseudomallei* has previously been reported^13,17,33,45^.

Based on an analysis of soil samples from southern US states, Hall et al.^46^ found *Burkholderia* sp. to be much more abundant in sandy soils than heavy in clay soils, hypothesizing that clay-based soils are more prone to anoxia which may limit the survival and growth of Burkholderiaceae. A recent study in Myanmar did not find any conclusive results of such an association between soil texture and *B. pseudomallei* due to a very low positivity rate^34^, while in peninsular Malaysia, Musa et al^24^ found that the odds of isolating *B. pseudomallei* were significantly higher for samples with higher clay content. Such apparent contradictions between reports further outlines the need for a description of the structural and pedomorphological features of soils layers that accounts for the depth-wise variability of soil physical and biogeochemical conditions and their complex interactions, beyond the mere consideration of soil types or of simple descriptors such as particle size distribution. In this study, even though *B. pseudomallei* preferentially occurred in clay-rich horizons, it must be noticed that these horizons also had a well-developed structure, as indicated by their low BD (hence high porosity) and high hydraulic conductivity at saturation.

### Groundwater level, groundwater persistence rate, water content, bulk density, hydraulic conductivity

Laboratory investigations showed that *B. pseudomallei* can survive about a year in soil with a moisture content of 20% while its survival is reduced to 30 days in dry soil^19^. Soil, at least near the ground surface, is a medium that is exposed to extreme variations in moisture content, depending on rainfall, air temperature and wind. While some studies reported higher rates of soil sample positivity for *B. pseudomallei* in the dry season compared to the rainy season^47^, previous environmental studies generally suggest that the presence of *B. pseudomallei* is associated with moist soils^8,9,48^ and groundwater. Several studies indicate that melioidosis is a disease that prevails in the rainy season ^2,49^, when the moisture of soil surface layers is high, increasing the likelihood of agricultural workers to be exposed to the organism, or when *B. pseudomallei* is discharged from naturally occurring seasonal groundwater seeps^50^.

At the time of sampling the soil profile analyzed in this work, which took place at the beginning of the rainy season, the soil water table was at a depth of 70 cm, and although *B. pseudomallei* was detected at almost all soil depths, bacteria counts were consistently higher below the depth of 110 cm. This depth of 110 cm is also the depth at which the groundwater persistence rate reaches 100%, indicating that below 110 cm soil was saturated with water all year-round. Such a co-occurrence of high *B. pseudomallei* counts with high soil moisture content is in agreement with results already obtained in Laos^16,51^.

Putative processes that could explain why *B. pseudomallei* is able to survive in dry soil and then become more abundant when the soil is rewetted could include survival in specific niches, locally differing in texture and water-holding capacity hence offering “micro-islands” of relatively wet soil within otherwise dry soil horizons. Another possibility suggested by others would be the upward migration of bacteria from a deep, round-year moist reservoir to shallower horizons, concomitantly with a rising water table^50,52^. It must however be noted that most bacterial transport is strongly correlated with solid particle transport (as the majority of bacteria are attached to solids) and that most solid transport processes in soil profiles are driven by infiltration under the effect of gravity.

BD measured along the soil profile varied substantially from soil surface to the depth of 290 cm. There was a strong negative correlation between *B. pseudomallei* counts and soil bulk density, indicating that *B. pseudomallei* occurred preferentially in parts of the soil that had a higher porosity (i.e., void/solid ratio). Soil density dropped abruptly from values of about 1.6 Mg m^−3^ to values of about 1.3 Mg m^−3^ between 110 and 130 cm, which, quite strikingly, coincides with the depth where both *B. pseudomallei* dramatically increases and where the groundwater persistence rate reaches 100%. Additionally, we found a significant correlation between *B. pseudomallei* counts and saturated hydraulic conductivity (Ks). Together with data of soil texture, bulk density and groundwater persistence rate, the correlation between *B. pseudomallei* counts and saturated hydraulic conductivity indicates that, at that location, *B. pseudomallei* was more abundant in deep clay silt soil layers with a porosity sufficient to allow for a good diffusion of water and gases. Indeed, it was also observed that the more porous horizons where *B. pseudomallei* prevails, between depths of 120–180 cm, are characterized by the presence of preferential flow pathways consisting mostly of biopores. Such an interpretation is corroborated by in situ measurements of dissolved oxygen (DO) at 210 cm (Fig. SI3): DO only dropped transiently below 0.5 mg l^−1^ (Fig. SI3), the threshold below which water is considered as anoxic according to Zogorski et al.^53^. Therefore, it can be assumed that oxic conditions, under which organisms can use oxygen for their metabolism, prevailed at the time of sampling at the soil depths where high concentrations of *B. pseudomallei* were observed.

It has been experimentally observed^54^ that the number of soil microorganisms declined linearly with increasing soil density from 1.00 to 1.60 Mg m^−3^. Different soil bulk densities correspond to different arrangements of the organic and inorganic constituents of soil, hence different types of porosity, the connectivity and tortuosity of which eventually governs the movement of fluids and associated solutes, particles and organisms, through soil^55^. Such an arrangement of pores and solids, referred to as soil structure, results in a diversity of niches with contrasted biogeochemical conditions, including substrate availability, hence harbouring diverse microbial communities^56,57^. In turn, metabolic processes associated with these microbial communities are one of the main drivers of soil structure and fertility formation and maintenance^58,59^.

### Physico-chemical factors

Our experimental results point out several correlations between soil physico-chemical parameters and *B. pseudomallei* counts.

Soil pH is known to be a strong predictor of soil bacterial community structure^60,61^. In this study, soil pH was acidic throughout the profile (average pH = 4.86), reaching values above 5 only near the soil surface (resulting in a strong negative correlation with *B. pseudomallei* counts). This result contrasts slightly with results from microcosm experiments that indicate better survival of *B. pseudomallei* within the 5 to 7 soil pH range, with survival reducing below pH = 4^62^. Overall, the genus *Burkholderia* appears to be acid-tolerant and field surveys of *B. pseudomallei* indicate that it is indeed most generally associated with low pH soils^15,63^.

The vertical distribution of *B. pseudomallei* counts in the soil profile was positively correlated with the total concentrations of iron, magnesium, potassium, aluminium, titanium, and negatively correlated with silica and calcium, which might mirror a correlation of *B. pseudomallei* counts with the mineralogical composition of different soil layers. Indeed, *B. pseudomallei* counts were also positively correlated with the finer textured soil material (clay and silt) and anticorrelated with coarse-textured material (sand), which corresponds to different minerals associations with different surface properties. X-ray diffraction investigations revealed that the granulometric clay fraction (i.e., < 2 µm) was mainly composed by a complex clay minerals assemblage (illite, Mg-Vermiculite, chlorite, kaolinite, and inter-stratified phases) associated to iron oxyhydroxides (goethite and lepidocrocite). In this granulometric fraction, quartz remained a minor phase although it largely dominated X-ray patterns for bulk soil samples, particularly for the sandiest horizons. Further mineralogical quantification would be needed to unravel this putative correlation between B. *pseudomallei* counts and the different mineralogical phases.

Previous field investigations indicated that, at a depth of 30 cm, the probability of finding *B. pseudomallei* in soil was higher when clay and iron contents were higher^24^, a result that these authors interpreted as being related to the water and nutrient retention properties of clay minerals, despite their tendency to increase waterlogged, hence anoxic conditions. Other authors found, based on replicate soil sampling at 30 cm depth, that *B. pseudomallei* is more common, in soils with low organic matter and nutrients contents, including phosphorus, potassium, calcium, magnesium, and iron^16^ and even that growth-limiting conditions such as nutrient and oxygen limitation can lead to formation of persister cells^64^.

Iron is known as an essential nutrient for most living organisms, including bacterial pathogens, as it is pivotal to many enzymatic and metabolic processes. *Burkholderia* species, including *B. pseudomallei*, are known to have evolved several iron uptake pathways, including the production of siderophores, and the ability to take up heme in infected hosts^65^, making them perfectly equipped to mobilize Ferric iron (Fe^3+^) the less bio-available but most common form of environmental iron in aerobic environments^7,66,67^. Reports regarding the association of *B. pseudomallei* with iron in soil are highly contradictory, with positive^24,58,66^ or negative correlations^17,68^ being seemingly equally likely. Yet, most reports did not include any indication of the bioavailability of iron or the oxidation state of the prevailing redox conditions of the environments in which such putative correlations were assessed. While known to be aerobic, *B. pseudomallei* can also survive anaerobiosis and stable subpopulations have been observed to survive under anaerobic conditions for at least one year, although growth was inhibited and metabolism most likely minimal^69^. In light of such findings, it is therefore quite possible that, in soils, depending on oxic conditions at the very local scale, *B. pseudomallei* switches metabolism and uses various mixes of ferrous and ferric iron, hence rendering the interpretation of correlations between the organism and total iron very uncertain—and to some extent, meaningless—in the absence of additional information. Indeed, in infected hosts, *B. pseudomallei* is able to cope with iron-restricted conditions by up-regulating its iron-acquisition system and use alternative metabolic pathways (i.e., other available electron donors/receptors) for energy production^23^.

In the case of the soil profiles studied in the work, we observed that there was a positive correlation between *B. pseudomallei* counts and several metallic element contents, including iron and fine textured soil material in association with a positive correlation with saturated hydraulic conductivity and organic matter content and a negative correlation with bulk density, while measured dissolved oxygen values indicated that strictly anoxic conditions rarely occurred at the soil depths where *B. pseudomallei* was the most abundant (Fig. SI3). Together, these observations suggest that, within a given soil profile, *B. pseudomallei* may primarily thrive in horizons where sufficient organic substrates, metallic elements (among which ferric iron) and moisture are available year-round, and where oxic conditions prevail, as a result of a soil structure (pore network of minimal connectivity and low tortuosity) sufficiently developed to allow minimal circulation of groundwater and supply of oxygen in dissolved form. This does not exclude, as previously reported, the simultaneous occurrence of other subpopulations of *B. pseudomallei*, in other soil horizons or subsets (niches) of the same soil horizons where overall conditions substantially differ from that where the bacteria was found to be the most abundant in this study.

Under field conditions, the lateral variability of biogeochemical properties within soil horizons may also potentially affect the vertical distribution of *B. pseudomallei* at any given point in time. We have checked by means of electrical resistivity tomography (ERT—Fig. SI4) that soil horizons were laterally very homogeneous over distances of ~ 35 m at our study site. Yet, it cannot be ruled out that *B. pseudo*mallei concentrations may vary substantially at shorter scales, within niches where biogeochemical conditions locally differ significantly from that that prevail on average within individual soil horizons.

Finally, it is noteworthy that the relevance of our results with regards to the occurrence, persistence and dispersal of *B. pseudomallei* in soil might also hold for other soil pathogens that can persist and disperse in groundwater (such as viruses).

### Limitations of the methodological approach

One major shortcoming of this study is the lack of internationally-validated methods for accurately detecting and quantifying *B. pseudomallei* in environmental samples such as soil or water. Molecular methods have generally given higher yields than culture-based methods and have the ability to detect bacteria that are in a viable but non-culturable state (as well as non-viable organisms), whilst cultural methods have varied considerably in their sensitivity in different studies^70^.

All detection methods lack precision and none will reliably determine which soil samples contain *B. pseudomallei*. We therefore chose to use a well-established semi-quantitative culture method for this study, rather than introducing a molecular assay which would have required additional validation in this setting.

More detailed typing of *B. pseudomallei* isolates was not conducted as part of the study but in view of our findings, further investigation may be warranted.

This work is based on a set of 45 soil subsamples that were collected once, and along a single vertical 300-cm soil profile. Obviously, this provides only a snapshot of what might happening over longer time scales. This is particularly important as it has been shown that the physico-chemical characteristics of soil water may vary considerably (Fig. SI3). However, an additional 300-cm vertical profile collected in December 2018 revealed depth-wise *B. pseudomallei* concentrations of the same order of magnitude as that of May 2018 (Fig. 2), at least below 100 cm, i.e. at depths where GPR was 100%. Although a more detailed study of the dynamics of the vertical distribution of the organism along the 300-cm profile is needed to assess whether seasonal variations in soil physico-chemical conditions alter the depth-wise concentrations of *B. pseudomallei*, this December 2018 observation indicates that 1. some degree of persistence of the *B. pseudomallei* concentrations in deep soil layers and 2. that reported results can be regarded with a good degree of confidence, despite the methodological limitations of the semi-quantitative approach used in this study.

## Conclusions

In this paper, we have presented vertical distributions of *B. pseudomallei* to a previously unexplored depth range of 300 cm. Previous reports of *B. pseudomallei* occurrence in deep bore water do not directly compare with our study as they did not attempt to elucidate the associated soil geochemical environment. The novelty of the results presented in this paper is that unravelling the environmental conditions favorable to *B. pseudomallei* presence and proliferation entails considering many aspects of the actual complexity of soil. This complexity includes not only soil chemical and textural characteristics, but also its hydrodynamic properties, its structural features nested over a wide range of scales, as well as its general arrangement in layers, the sequence of which forms the soil profile. As a direct consequence of the work presented in this paper, it can be reasonably assumed that the odds of isolating *B. pseudomallei* in dry environments would often been higher from deep than shallow soil layers. Finally, even though this point was only very marginally considered in this study, the inherently dynamic nature of the complex biogeochemical interactions that constantly transform the soil environment must also be taken into account, as transient changes in parameters of central importance for the metabolism of microorganisms, such as dissolved oxygen, can vary drastically within a matter of hours under the influence of weather conditions.

As it sheds new light on how *B. pseudomallei* is vertically distributed within a soil profile, including at depths generally not taken into account in environmental *B. pseudomallei* studies, this work also identifies new potential hazards related to human activities that involve interactions with deep soil layers. This is potentially clinically significant because human activities have become the main geomorphological process on the Earth's surface, disturbing and displacing globally ten times more soil, sediment and rock material than natural geological processes^71^. Hence, beyond agriculture, which generally involves no or limited interactions between humans and deep soil layers, earthworks across the globe are now dramatically increasing chances of people being in contact with material extracted from several meters under the soil surface, potentially hosting pathogenic bacteria, as shown in this study.

Key recommendations from this work regarding the environmental sampling of *B. pseudomallei* include:

1. collecting samples at successive depth increments from the soil surface, as many soil properties vary much more drastically vertically than horizontally. Hence a single soil profile maximizes the odds of detecting *B. pseudomallei* at a given location compared to the same number of samples taken at a single soil depth;
2. collecting samples down to the depth at which the water table is found at the time of sampling, as groundwater persistence appears to be an important factor for the presence and possibly persistence of *B. pseudomallei*;
3. recording basic pedological information such as soil colors and macrostructural features (redoximorphic features), as these simple observations provide valuable information about the redox conditions at the point of sampling.

Despite the significant advances that this study brings for a better understanding of the ecology of *B. pseudomallei*, it still leaves important questions unaddressed such as, in particular the influence of: (1) time variability of soil conditions, and (2) the variability of soil conditions at very local scales (mm and less) on the persistence and proliferation of *B. pseudomallei.* It also remains to be clarified whether *B. pseudomallei* colonies that occur in association with different pedofeatures along a soil profile such as that studied in this work pertain to one single or to different strains.

## Materials and methods

### Study site

Soil sampling was conducted in a lowland paddy rice field (18° 22′ 59.02″ N, 102° 25′ 22.02″ E; altitude 180 m above mean sea level) in Nabone village (Fig. 1), Phonhong district, Vientiane Province, Laos, in an area where *B. pseudomallei* had been detected during previous studies^16,72^. Sample collection was conducted following the approval of provincial administration of agriculture and forestry and with the agreements of both the land owner and the chief of the village.

The area experiences a tropical monsoon climate with three main seasons: a rainy season from May to October, a cool dry season from November to February and a hot dry season from March to April. Over the 2010–2016 period, average annual rainfall at nearby Phonhong station was 1519 mm and mean annual temperature was 27.4 °C (Department of Meteorology and Hydrology, Laos).

Geologically, the studied area is located in the northern margin of the Khorat plateau basin and belongs to the Vientiane sub-basin, a topographic plain that is an extension of the Upper Cretaceous Sakhon Nakhon basin. Rocks in the area consist of Cretaceous continental deposits (Saysomboun formation), mainly red-brown fluvial claystone to fine sandstone sedimentary formations partially covered by quaternary fluvial deposits containing gravel, sand and clays^73^. Soils that develop over these sedimentary formations are mainly Acrisol (ferric Acrisols, gleyic Acrisol and haplic Acrisols) and are for the most cultivated (mainly rice based cropping system).

### Soil sampling and field observations

At the onset of the 2018 rainy season (early in May), a motorized percussion corer (Cobra TT; 14.19SD HM1400—https://www.atlascopco.com) was used to collect three soil cores (100 cm long, 10 cm inner diameter), so as to sample an overall depth of 300 cm below the soil surface (Fig. 3). Every 20 cm, from 10 to 290 cm below the soil surface, undisturbed triplicate 40 cm^3^ soil sub-samples were taken from the soil cores with small PVC cylinders. In total, 45 subsamples were collected along the vertical soil profile. Chemical and mineralogical analyzes were performed on air-dried samples. At each soil depth increment, 100 g of soil was also taken for subsequent *B. pseudomallei* determination. These samples were temporarily stored in sterile plastic bags, sealed and placed in a cool box in the shade prior to being transported to the laboratory within 6 h. Sampling instruments were cleaned after each sample collection, using water to remove soil and subsequently desinfected with 70% ethyl alcohol.

Morphological characteristics of soil horizons were observed and described in the field (Fig. 3), immediately after core extraction, in order to avoid re-oxidation of samples taken from horizons where reducing conditions may prevail. These morphological characteristics include: soil matrix color, soil structure and macroporous features, reductimorphic and redoximorphic features^74^, mottled colors, identification of clay leaching and iron oxide nodules pedofeatures (as indicators of clay and iron dynamics). Soil color was estimated using the Munsell colors chart^37^.

From the beginning of May 2018 to the end of April 2019, selected physico-chemical properties of the soil solution (i.e. water with dissolved gases, minerals, and organic matter that make up the liquid phase of soil) were monitored monthly, directly in a 210 cm deep piezometer using an YSI 556 MPS multi-parameter probe (www.ysi.com); measured parameters were: temperature, electrical conductivity (EC), dissolved oxygen (DO), pH and oxidation–reduction potential (ORP) (Fig. SI3).

An additional 300-cm vertical profile was collected in December 2018, following the end of the rainy season, for the purpose of assessing depth-wise *B. pseudomallei* concentrations only. This second profile was sampled at a distance of < 5 m from the location of the May 2018 profile. No further pedological nor physico-chemical characterization was attempted on the basis of this second sampling. Rather, this sampling was intended to assess whether the order of magnitude of the semi-quantitative estimates of *B. pseudomallei* varied substantially at the seasonal scale.

### Soil physico-chemical parameters

Soil water content (WC), bulk density (BD), pH, organic matter content (OM) and texture were measured at each sampling depth. WC and BD were measured using intact 40 cm^3^ soil cores collected with small PVC rings; moist soil cores were first weighed, then oven dried at 105 °C for 48 h, then reweighed; WC is calculated as the ratio of the mass of water lost by drying divided by the mass of dry soil whereas BD is the ratio of the mass of dry soil divided by the volume of the core^75^. For soil pH, 20 g of soil was diluted into 50 ml of deionised water and the pH of this solution was subsequently measured with a Hanna (Woonsocket, RI, USA) laboratory pH-meter. The standard Walkley–Black operating procedure was used for OM determination^76^. Soil texture (i.e. Clay < 2 μm; Silt 2–50 μm; Sand 50–2000 μm) was analyzed at each sampling depth following the standard pipette method^77^.

### Soil elemental concentrations and mineralogy

Elemental concentration measurements and mineralogical properties of soil samples were performed at the Geosciences Environment Toulouse (GET) laboratory, Toulouse, France. Soil elemental concentrations of major elements (Na, Mg, Al, Si, P, S, K, Ca, Ti, Mn and Fe) were determined on 1 g of finely ground powder samples fused with 10 g of Li_2_B_4_O_7_:LiBO_2_ (mass ratio of 66:34) into Pt crucibles to produce glass discs for X-ray fluorescence analysis by the way of a Bruker S2 Ranger energy-dispersive X-ray fluorescence analyser, using Pd X-ray tube and Peltier-cooled silicon drift detector. Measurements were run with the X-ray tube successively tuned to 20, 40 and 50 keV.

The mineralogical composition of soil samples (bulk samples and clay fraction) was determined using X-ray diffraction (XRD). XRD measurements for random powder analysis were performed on the Bruker D2 diffractometer (Cu-Kα radiation, Brag Brentano theta/theta setup, 2°–80°) after crushing the total fraction in an agate mortar. X-ray diffraction measurements were made on the clay granulometric fraction (0–2 µm) from oriented samples for 6 selected samples (at 10, 30, 90, 170, 210 and 250 cm depth) and were performed using D8 Advance (www.bruker.com) diffractometers (Cu-Kα radiation, Brag Brentano theta/theta setup, 2°–30°). Diffraction patterns of powder samples were interpreted with reference to the ICDD database (PDF-2^TCM^) and COD databases using EVA software (Bruker).

Exchangeable Ca^2+^ (Ca_e), Mg^2+^ (Mg_e), Na^+^ (Na_e) and K^+^ (K_e) concentrations of soil were estimated using the 1 N ammonium acetate method^78^. Cation exchange capacity (CEC) of this soil and exchange acidity (EA) were estimated based on calculations considering the “sum of base cations” and the “sum of acid cations” approach using both pHKCl and pHH_2_O values^79^.

### Microbiological analysis

*Burkholderia pseudomallei* was isolated from soil as as previously described^16,72^. Briefly, 100 g of each soil sample was added to 100 ml of sterile de-ionised water, and mixed well by agitation, for semi-quantitative culture of *B. pseudomallei* on Ashdown agar plates^70^. After overnight sedimentation at room temperature, the supernatant was transferred to a separate sterile container. Supernatant aliquots in duplicate of 10, 100 and 500 µl were plated and spread onto Ashdown’s agar, incubated at 42 °C in air, and inspected for up to 4 days. Suspect colonies (by morphology) were identified as presumptive *B. pseudomallei* if they were colistin and gentamicin resistant, co-amoxiclav susceptible and specific latex agglutination-positive. The number of presumptive *B. pseudomallei* colonies on each plate was recorded and colony counts calculated. A subset of presumptive *B. pseudomallei* isolates were confirmed by API20NE (bioMérieux) phenotypic testing. The only other *Burkholderia* sp. that might have caused confusion is *B. thailandensis*, that expresses *B. pseudomallei*-like capsular polysaccharid (CPS; known as BTCV). Such isolates have been found frequently in Thailand, although only once in Laos^80^. This is why a subset of isolates were tested by API 20NE, as these two organisms can be distinguished by arabinose assimilation (*B. pseudomallei* being negative while *B. thailandensis* is positive). No BTCV were found.

### Groundwater level monitoring and hydrodynamic properties of soil

Groundwater was monitored using a 210 cm deep piezometer installed in the field within less than 5 m of the location where the soil core has been collected. A water level pressure sensor (CTD-Diver datalogger), submerged at the base of the piezometer, was used to automatically record the pressure at 15-min intervals during one year, starting from early May 2018. Water levels were derived from these measurements after correcting the variations in atmospheric pressure using data from a barometric probe (Baro-Diver datalogger) installed next to the piezometer. Manual groundwater level measurements were also taken using this piezometer, monthly and bi-monthly during the dry and rainy seasons, respectively (Fig. SI3).

Saturated hydraulic conductivity (Ks) of soil was measured in the field at 5 depth increments (namely 10, 50, 90, 130, and 170 cm), in 6 replicate test boreholes, using a Wiltschut permeameter^81^. These measurements were conducted in the vicinity (at a distance of < 2 m) of the soil profile sampled for *B. pseudomallei* and associated physico-chemical parameters*.*

### Data analysis

As some datasets were not normally distributed, a Spearman’s rank-order correlation, also known as a non-parametric measure of rank correlation, was applied (XLSTAT Premium version 20.1.1.) for all data analysis and test putative interactions between semi-quantitative estimates of *B. pseudomallei* in samples with measured soil properties.

We conducted a Partial Least Square Regression (PLS-R) analysis using the natural logarithm of the semi-quantitative colony forming unit (CFU) counts of *B. pseudomallei*, and physico-chemical parameters determined at all sampled soil depths along the 300-cm deep profile (XLSTAT Premium version 20.1.1.). PLS-R is well-suited to the analysis of a dataset with few observations and many variables such as that corresponding to the 300-cm soil profile studied in this work. The variable importance in the projection (VIP) number was used to assess the relative importance of projected variables, with variables having a VIP < 1 considered to be unimportant in the analysis^13^.

## Supplementary Information

Supplementary Information.

## Data Availability

The datasets generated and analysed during the current study will be available on the DataSuds repository: https://doi.org/10.23708/L3IJGD.
