# Supplementary material for: Distribution of Burkholderia pseudomallei within a 300-cm deep soil profile: implications for environmental sampling
Source: Sci Rep. 2022 May 23;12:8674. doi: 10.1038/s41598-022-12795-0 (PMC9126866; doi:10.1038/s41598-022-12795-0)
Supplement: Supplementary file 1 — Supplementary Information. [file 41598_2022_12795_MOESM1_ESM.pdf]

Supplementary Information

Distribution of *Burkholderia pseudomallei* within a 300-cm deep soil profile – implications for environmental sampling

Khemngeun Pongmala[a,b], Alain Pierret[c], Priscia Oliva[a], Anne Pando[c], Viengmon Davong[d], Sayaphet Rattanavong[d], Norbert Silvera[c], Manophab Luangraj[d], Laurie Boithias[a], Khampaseuth Xayyathip[c], Ludovic Menjot[a], Melina Macouin[a], Emma Rochelle-Newall[e], Henri Robain[e], Amphone Vongvixay[f], Andrew J H Simpson[d,g], David AB Dance[d,g,h], Olivier Ribolzi[a]*

[a]GET, Université de Toulouse, CNRS, IRD, UPS, Toulouse, France

[b]Faculty of Environmental Sciences, National University of Laos, Vientiane, Lao P.D.R.

[c]Institut de Recherche pour le Développement (IRD), IEES-Paris UMR 242, Sorbonne Université c/o Department of Agricultural Land Management (DALaM), Vientiane, Lao P.D.R.

[d]Lao-Oxford-Mahosot Hospital-Wellcome Trust Research Unit (LOMWRU), Microbiology Laboratory, Mahosot Hospital, Vientiane, Lao PDR

[e]Institute of Ecology and Environmental Sciences of Paris (iEES-Paris), Sorbonne Université, Univ Paris Est Creteil, IRD, CNRS, INRA, 4 place Jussieu, 75005 Paris, France

[f]Faculty of Engineering, National University of Laos, Vientiane, Lao P.D.R.

[g]Centre for Tropical Medicine and Global Health, Nuffield Department of Medicine, University of Oxford, Oxford, United Kingdom

[h]Faculty of Infectious and Tropical Diseases, London School of Hygiene and Tropical Medicine, London, United Kingdom

* Address correspondence to Olivier Ribolzi

Email: olivier.ribolzi@ird.fr

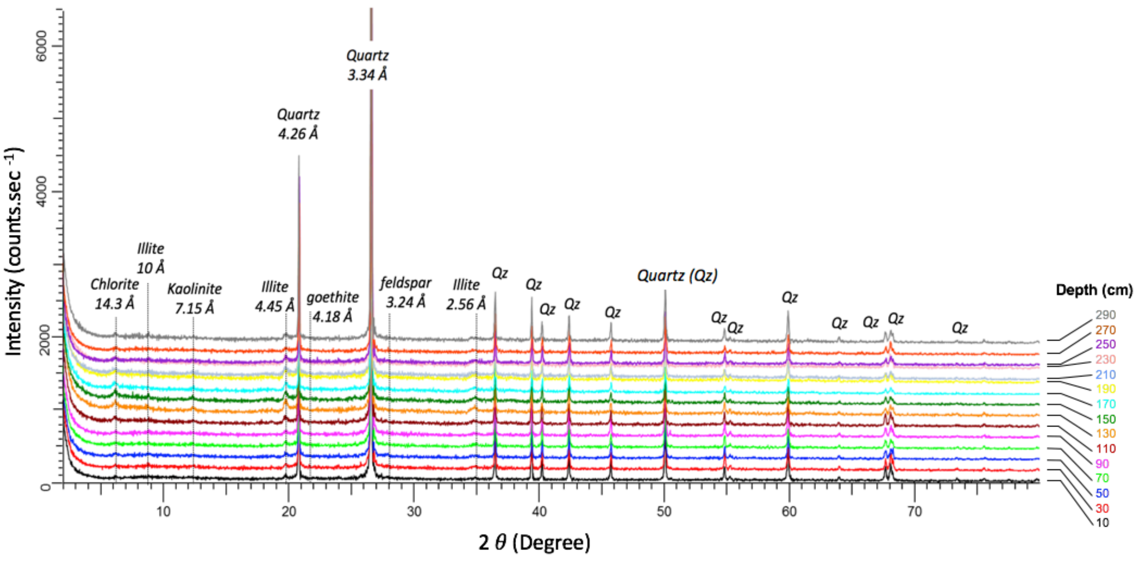

Figure SI 1 X-ray diffractograms (XRD) of the bulk soil samples collected every 20cm along the 300-cm deep soil profile. XRD patterns show the high intensity peaks of quartz (Qz), chlorite, illite, kaolinite, felspar and goethite, quartz being the major mineralogical phase for all bulk soil samples. Mineralogical analysis were performed at the Geosciences Environment Toulouse (GET) laboratory, Toulouse, France. XRD measurements for random powder analysis were performed on a Bruker D2 diffractometer (Cu-Kα radiation, Brag Brentano theta/theta setup, 2-80°) after crushing the total fraction in an agate mortar. DIFFRAC^{plus} EVA software and the ICDD PDF-2 Database helped for the identification of minor and major mineralogical phases.

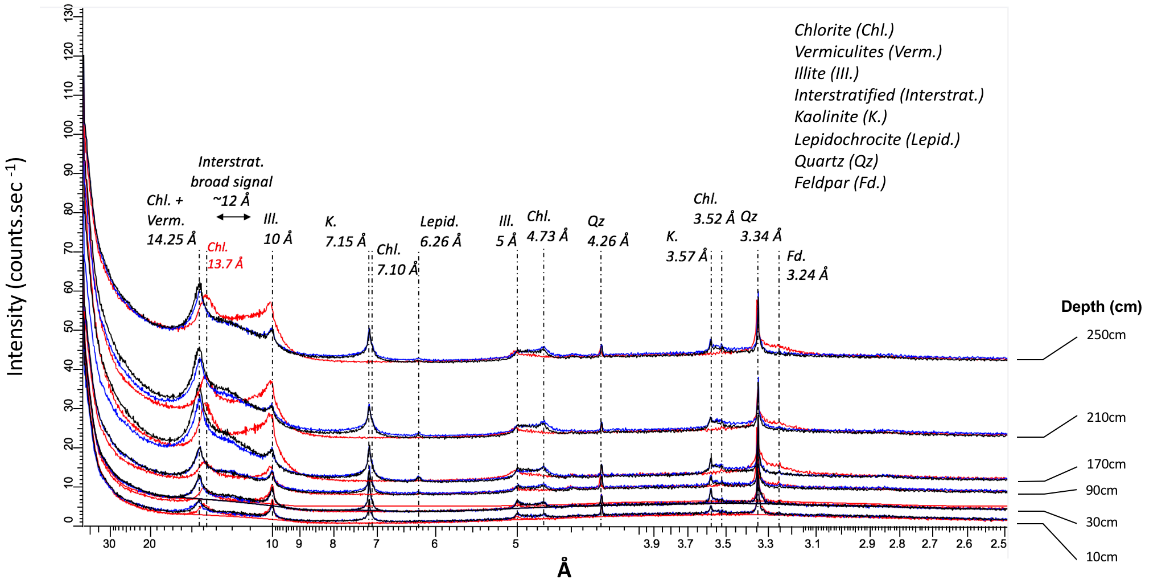

Figure SI 2 X-ray diffractograms on oriented clay granulometric fraction (0-2μm) of 6 selected soil samples (at 10, 30, 90, 170, 210 and 250 cm depth) along the 300-cm deep soil profile. Clay minerals were identified using the basal d-spacing reflections as identified using XRD. They were differentiated by comparing the XRD patterns obtained under three different types of preparation: air dried (black pattern), after glycolation with éthylène glycol-EG (blue pattern) and after heating at 500°C (red pattern). Oriented clay slides were prepared for XRD analyses by pipetting the suspended clay fractions onto petrographic slides and allowing the slides to dry overnight for the air-dried and the EG treatments. On the different XRD diffractograms, the peak at 14.25 Å was attributed to chlorite and vermiculite. The displacement of the 14.25 Å peak to 13.7 Å after heat treatment can be attributed to chlorite and the parallel increase of the peak at 10 Å and the disappearance of the broad reflection at ~12 Å are indicative of vermiculite and interstratified phases with vermiculite layers. Kaolinite was identified by the 7.15 Å and 3.57 Å peaks that deseapear after heating at 500°C. Illite showed a 10 Å reflection in all

treatments. Non clay phases such as quartz, felspar and lepidocrite were also found in oriented <2µm fractions of the studied soil samples. Mineralogical properties were performed at the Geosciences Environment Toulouse (GET) laboratory, Toulouse, France using X-ray diffraction (XRD). XRD were performed on oriented samples using D8 Advance (www.bruker.com) diffractometers (Cu-Kα radiation, Brag Brentano theta/theta setup, 2-30°).

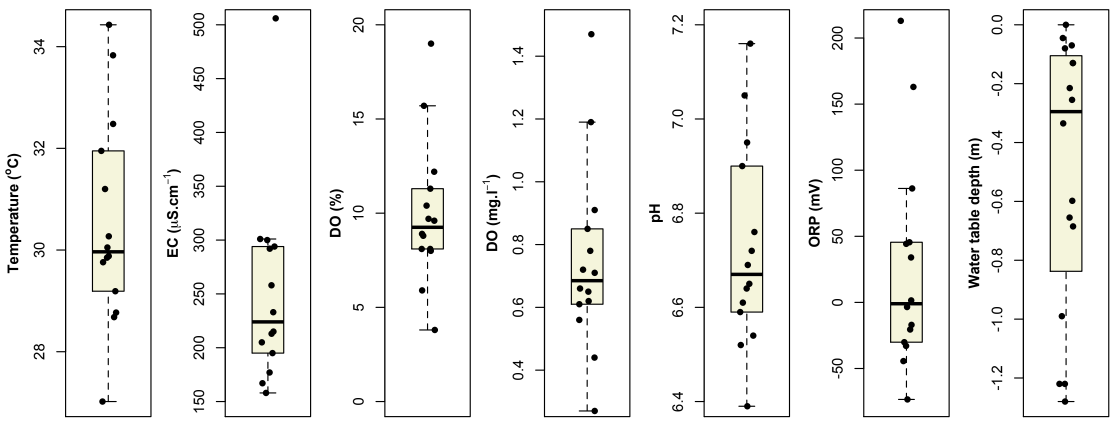

Figure SI 3. Box plots of physico-chemical parameters measured in the groundwater using a 210 cm deep piezometer: temperature ; electrical conductivity (EC); disssolved oxygen (DO); pH ; oxydo-reduction potential (ORP) ; Water table depth. The central horizontal line indicates the median values, and the upper and lower edges of the boxes (hinges) indicate the 25th and 75th percentile values, while the whiskers extend 1.5 × the spread of the hinges. Data points outside this range are indicated with circles.

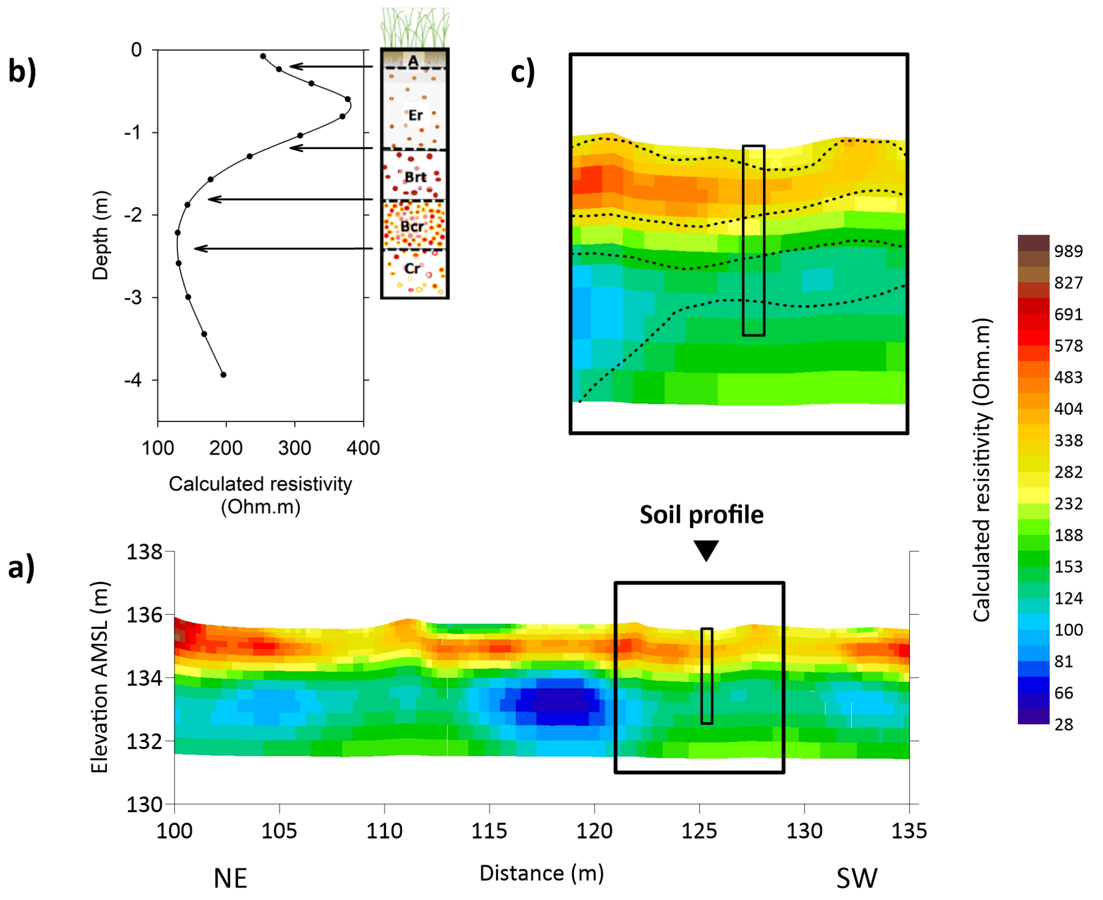

Figure SI 4. 2D model of ground electrical resistivity calculated from the geophysical survey carried out in december 2018. Field measurements were performed along a linear profile with a multi-electrode resistivitymeter (SYSCAL Pro 72 equipment, Iris Instruments) and 0.5 m unit inter-electrode spacing. The configuration of measurement arrays was reciprocal Wenner-Shlumberger (Szalai and Szarka, 2008). The field data set was processed using RES2DINVx64 software (Geotomo Software, Malaysia) to calculate a 2D model of electrical resistivity distribution within the ground. The model had 14 layers of parallelepipedic blocs (homogeneous width 0.25 m and thichness increasing of 10% for each layer from 0.12 m for the shallowest one until 0.59 for the deepest one. The total depth of investigation was 4.18 m.

(a) 35 meters long 2D ERT profile crossing the location of the soil profile; **(b)** Vertical log of calculated resistivity at soil profile location and picking of values corresponding to observed interfaces along soil profile **(c)** Zoom (8m wide) around the soil profile (see schematic of the soil log, Fig. 3).

The ERT profile reveals 5 layers with contrasted electrical resisitivity which are consistent with the vertical succession of the main soil horizons (ie A, Er, Brt, Brc and Cr). Their lateral extension in the area could thus be approximated following the method described in Ribolzi et al. (2018). Significant lateral variations of thickness and electrical resistivity (probably corresponding to clay/sand content variations, e.g. Shevnin et al. et al, 2007) appears. Nevertheless the vertical succession of the 5 horizons observed along the soil profile is not changing laterally.

References cited in the legend of figure SI 4

Szalai S. and Szarka L, 2008. On the classification of surface geoelectric arrays. Geophysical Prospecting, 56, 159–175 https://doi:10.1111/j.1365-2478.2007.00673.x.

Shevnin V., Mousatov A., Ryjov A. and Delgado-Rodriquez O. 2007. Estimation of clay content in soil based on resistivity modelling and laboratory measurements. Geophysical Prospecting, 2007, 55, 265–275

Ribolzi, O., Lacombe, G., Pierret, A., Robain, H., Sounyafong, P., de Rouw, A., Soulileuth, B., Mouche, E., Huon, S., Silvera, N., Latxachak, K.O., Sengtaheuanghoung, O., Valentin, C., 2018. Interacting land use and soil surface dynamics control groundwater outflow in a montane catchment of the lower Mekong basin. Agric. Ecosyst. Environ. https://doi.org/10.1016/j.agee.2018.09.005
